# Novel treatments in optic pathway gliomas

**DOI:** 10.3389/fopht.2022.992673

**Published:** 2022-09-29

**Authors:** Akash Maheshwari, Mohammad Pakravan, Chaow Charoenkijkajorn, Shannon J. Beres, Andrew G. Lee

**Affiliations:** ^1^ Department of Ophthalmology, Blanton Eye Institute, Houston Methodist Hospital, Houston, TX, United States; ^2^ School of Medicine, Texas Tech University Health Sciences Center, Lubbock, TX, United States; ^3^ Department of Ophthalmology, Baylor College of Medicine, Houston, TX, United States; ^4^ Department of Neurology and Neurosciences, Stanford University, Palo Alto, CA, United States; ^5^ Department of Ophthalmology, Stanford University, Palo Alto, CA, United States; ^6^ Department of Ophthalmology, Weill Cornell Medicine, New York, NY, United States; ^7^ Department of Neurology, Weill Cornell Medicine, New York, NY, United States; ^8^ Department of Neurosurgery, Weill Cornell Medicine, New York, NY, United States; ^9^ Department of Ophthalmology, University of Texas Medical Branch, Galveston, TX, United States; ^10^ Department of Ophthalmology, University of Texas MD Anderson Cancer Center, Houston, TX, United States; ^11^ Department of Ophthalmology, Texas A and M College of Medicine, Bryan, TX, United States; ^12^ Department of Ophthalmology, The University of Iowa Hospitals and Clinics, Iowa City, IA, United States

**Keywords:** optic pathway glioma, low grade glioma, chemotherapy, immunotherapy, MEK inhibitor, BRAF inhibitor, bevacizumab, stereotactic radiation

## Abstract

Optic pathway gliomas (OPG) are primary tumors of the optic nerve, chiasm, and/or tract that can be associated with neurofibromatosis type 1 (NF1). OPG generally have a benign histopathology, but a variable clinical course. Observation is generally recommended at initial diagnosis if vision is stable or normal for age, however, treatment may include chemotherapy, radiotherapy, or surgery in select cases. This manuscript reviews the literature on OPG with an emphasis on recent developments in treatment.

## Introduction

Optic pathway glioma (OPG) is a primary tumor of the optic pathway (e.g., one or both optic nerves, the optic chiasm, the optic tracts, and/or optic radiations) primarily found in pediatric patients. Contiguous spread to the hypothalamus can occur in OPG (1, 2). A significant amount of OPG patients have Neurofibromatosis type 1 (NF1) which often influences the severity and course of tumor growth. Although observation for clinical or radiographic progression is generally recommended initially for OPG (especially with NF1), some patients require therapy if there is progressive or significant visual loss. For many years, treatments have included chemotherapy and radiation therapy, but newer immunotherapies have emerged for OPG. This manuscript reviews the current status of these novel and emerging therapies in the treatment of OPG.

## Methods

A PubMed English language literature search was performed using the following search terms for the years 2010-2022: optic pathway glioma (OPG) and treatment, management, chemotherapy, immunotherapy, radiotherapy, stereotactic radiosurgery, stereotactic radiotherapy, tumor markers, tumor mitogen-activated protein kinase (MAPK) inhibitor, mitogen-activated protein kinase kinase (MEK) inhibitor, b-rapidly accelerated fibrosarcoma kinase (BRAF) inhibitor, and bevacizumab chemotherapy. Selected articles prior to 2010 were included for completeness or for background purposes. A total of 85 articles were included. Case reports, letters to the editor, and photo-essays were only included if the article added significant new information to the literature review. Duplicate citations were also excluded.

## Case

**Figure 1:**
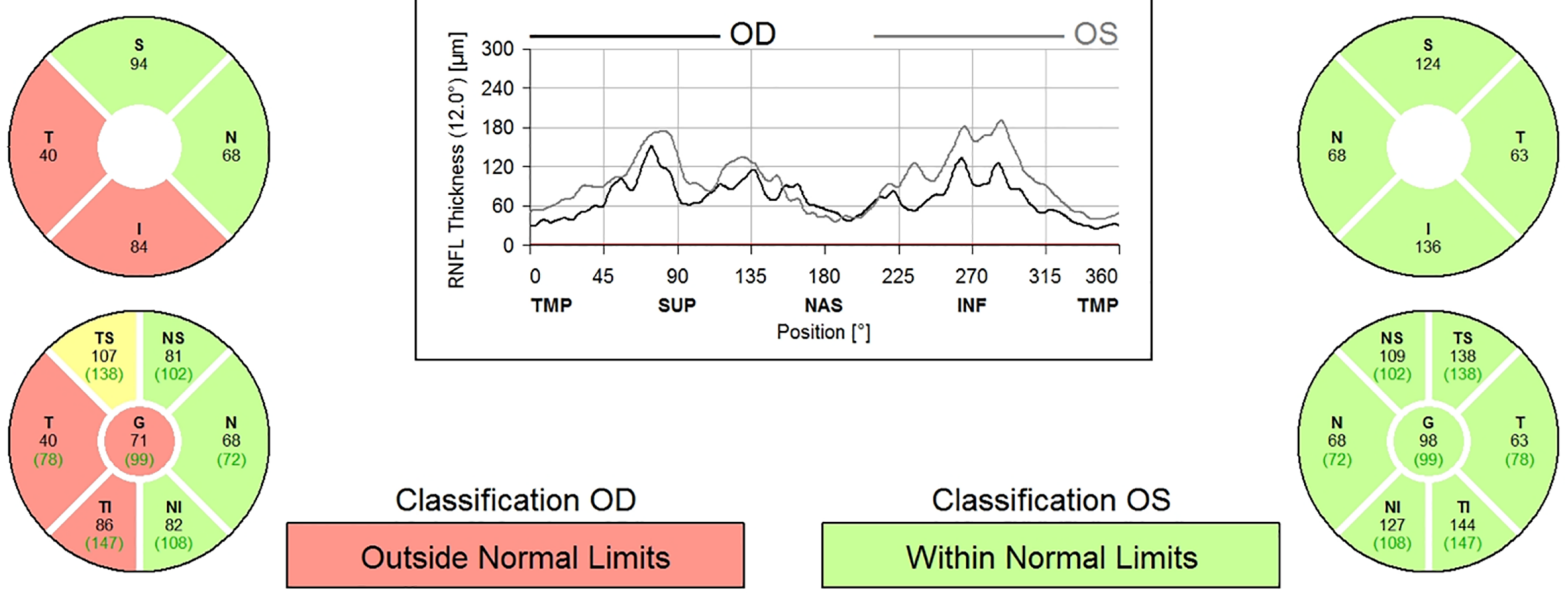
OCT measurement of RNFL thickness.

A 9-year-old boy with neurofibromatosis type 1 (NF1) presented to the ophthalmology clinic for his first eye exam after being lost to follow up since infancy with vision in the right eye (OD) of 20/25 and normal left eye (OS) vision. There was a relative afferent pupillary defect (RAPD) and mild optic nerve atrophy of the OD. Humphrey visual field (HVF) revealed a superior arcuate and paracentral defect OD but was normal OS. Optical coherence tomography (OCT) confirmed secondary loss of optic nerve fibers and a reduction in retinal nerve fiber layer thickness to 71 microns OD and 98 microns in OS (**Figure 1**). Magnetic resonance imaging (MRI) of the brain and orbit with contrast showed enlargement of the optic nerves with mild enhancement of the intraorbital portion of the optic nerve OD consistent with bilateral optic nerve gliomas without intracranial extension. See **Images 1**-**3**.

The patient was initially observed for 3 months as the onset of the vision loss was unclear and could have been long-standing, but on close interval follow-up the vision decreased (visual acuity 20/30 and HVF with increased mean deviation and arcuate scotoma OD) and repeat MRI showed increased enhancement of the right optic nerve mass. After consultation with the parents, he was treated with vincristine and carboplatin-based chemotherapy. One year after treatment, the MRI was stable and vision decreased to 20/40 OD. Serial imaging showed optic nerve glioma on the right eye had increased in size. A biopsy of the mass revealed BRAF mutation and he was started on selumetinib. On repeat MRIs over the next 1.5 years, glioma remained stable with no further growth while on selumetinib. His vision improved to 20/30 OD and left eye remained 20/20. OCT RNFL showed progressive thinning in both eyes.

## OPG presentation

The clinical presentation of OPG is dependent on location (e.g., one or both optic nerves, the optic chiasm, the optic tracts, and/or the optic radiations) as well as adjacent structures (e.g., hydrocephalus, hypothalamus). Although many cases of OPG are asymptomatic (3–5), especially in patients with NF1 (6), OPG can be vision or even life threatening (e.g., secondary obstructive hydrocephalus, endocrinopathy, diencephalic or hypothalamic involvement).

The anatomic location of the tumor is the major determining factor for the presenting symptoms and signs of OPG. Patients with an OPG confined to the optic nerve present with ipsilateral visual loss, a relative afferent pupillary defect, and a pale or swollen optic nerve (6, 7). Patients with intraorbital OPG may also have proptosis and/or strabismus. Intracranial OPG can present with hypothalamic endocrinopathy, spasmus nutans, obstructive hydrocephalus, or diencephalic syndrome (3, 4, 8, 9). The most common clinical presentation however is painless, progressive unilateral or bilateral visual loss (4) but diplopia (ophthalmoplegia), ptosis, nystagmus (spasmus nutans), or proptosis may also occur (5, 10). The screening protocol for pediatric patients presenting with potential OPG symptoms typically involves subsequent neuroimaging (including CT or MRI) and/or biopsy of the lesion.

Patients with NF1 related OPG have significant differences in presentation and prognosis compared to those without NF1 (“sporadic OPG”) and are more often asymptomatic. Patients with NF1 related OPGs who are symptomatic may present with higher frequency with proptosis and lower frequency with nystagmus and hydrocephalus (11). As a result, pediatric patients diagnosed with a NF1 gene mutation are advised to have complete eye examinations annually when under 10 years of age, and at least every 2 years until 18 years of age (12, 13). Age-appropriate visual acuity testing is essential for NF1-associated OPG surveillance, especially because visual field testing is often unreliable in pediatric patients (14). Recently, optical coherence tomography (OCT) has been found to be an objective modality in the observation of NF1-associated OPG (12, 14). OCT measures retinal nerve fiber layer (RNFL) thickness, recording secondary loss of optic nerve fibers, a reliable marker for visual loss in NF1 patients (12, 14).

## OPG diagnosis

### Imaging

Patients with signs and/or symptoms suggestive of an OPG including afferent (optic nerve, chiasm, or optic tract) or efferent neuro-ophthalmic involvement with or without endocrine manifestations should undergo neuroimaging. Magnetic resonance imaging (MRI) of the brain and orbit with gadolinium contrast is the preferred imaging modality for OPG (10). Computed tomography (CT) scans may be useful for bony structures and for identification of any calcification within the tumor (which can be indicative of low-grade histology), but CT involves radiation exposure which may have deleterious long-term effects in children. MRI is superior to CT for soft tissue resolution and for defining the extent of OPG involvement of the optic pathways (15).

On MRI, OPG are generally hypointense to isointense on T1 and hyperintense on T2 weighted images (4, 5) (**Image 4**). OPG typically show variable contrast enhancement (4) (**Image 5**). The imaging of OPG in NF1 patients is more likely to show bilateral involvement and extension to the optic chiasm and/or optic tracts. Involvement of the lateral geniculate body, hypothalamus, or temporal lobe may also occur (16). Non-NF1 patients with OPG more often have fusiform lesions (5, 17–20) confined to the optic nerve or optic chiasm, but can have more extensive spread. Surveillance imaging is needed with orbital and brain MRI to monitor radiographic changes to the OPG and treatment effects if undergoing therapy.

### Biopsy

Most cases of OPG can be diagnosed based on the clinical and radiographic appearance of the lesion. A diagnostic biopsy, however, provides a definitive diagnosis of OPG and World Health Organization (WHO) tumor classification subtype (1, 9) as well as other tumor markers which can be informative for treatment options. In the WHO tumor classification, WHO Grade I and II gliomas are tumors that progress slowly and are together known as “low-grade gliomas,” while WHO Grade III and IV gliomas are faster growing tumors known as “high-grade gliomas” (1). Most OPGs are WHO Grade I gliomas, although they are occasionally WHO Grade II gliomas. It is extremely rare for OPGs to progress to WHO Grade III or Grade IV gliomas (1).

WHO Grade I gliomas are the least aggressive tumors and have few characteristics of anaplasia (21). Pilocytic astrocytomas comprise the majority of low-grade OPGs and histologically have a biphasic pattern, characteristic Rosenthal fibers, and eosinophilic granular bodies (5, 9). Immunohistochemically, pilocytic astrocytomas are diffusely and strongly positive for GFAP and transcription factor Olig-2 (5). The prognosis of these tumors is typically very good with overall survival rates for OPGs (mostly pilocytic astrocytomas) after 10 years of 90% or more (22, 23). Most studies on OPG however do not differentiate survival rates between the different grades/types of gliomas, and treatment options will also vary the survival rate.

WHO Grade II gliomas are characterized by the following histopathology features related to the degree of anaplasia: cellular density moderately increased, occasional nuclear atypia, mitotic activity absent or 1 mitosis, necrosis absent, endothelial proliferation absent (21). WHO Grade II OPGs are either pilomyxoid astrocytomas or fibrillary astrocytomas and each has distinct characteristics. Pilomyxoid astrocytomas are composed of piloid cells in a loose fibrillary and myxoid background. These tumors are considered to be more aggressive than pilocytic astrocytomas and immunohistochemically label strongly and diffusely for GFAP and vimentin but are negative for the neuronal markers synaptophysin, neurofilament, chromogranin and epithelial membrane antigen (5). Komotar et al. reported that 14% of patients with pilomyxoid astrocytomas presented with CSF dissemination (24). Fibrillary astrocytomas (also known as diffuse astrocytomas) are well differentiated and characterized by a high degree of infiltration of neuropils (5). Neurofilament protein stains are helpful in histological viewing of infiltrative nature of this tumor (5). In general, the prognosis is worse for Grade II gliomas than Grade I gliomas, although OPG specific data in this area is limited (25).

Malignant OPG are extremely rare and are more often observed in adults than in children. One review reported fewer than 50 total cases of malignant OPG in 2014 (26). High grade gliomas are either anaplastic astrocytomas (Grade III) or glioblastomas (Grade IV). The prognosis for malignant OPG is poor and most patients die within a year of diagnosis (27, 28).

## Current management

The natural history of OPG is highly variable and depends on a multitude of factors. Asymptomatic or visually stable patients with OPG can be observed clinically with surveillance ophthalmologic exams and serial MR imaging (29). The first line of medical treatment for symptomatic and progressive OPG is often vincristine and/or carboplatin-based chemotherapy (30, 31). Other less commonly used chemotherapeutic agents include cisplatin, etoposide, and, temozolomide (9). Some of the common side effects associated with these agents are found in **Table 1**.

**Table 1:** Chemotherapeutic agents and side effects.

Chemotherapeutic Agent	Common Side Effects
Carboplatin	• Infection, especially when white blood cell count is low • Bruising, bleeding • Anemia which may cause tiredness, or may require blood transfusions • Vomiting, nausea • Hair loss
Cisplatin	• Infection, especially when white blood cell count is low • Bruising, bleeding • Anemia which may cause tiredness, or may require blood transfusions • Kidney damage which may cause swelling, may require dialysis • Hearing loss including ringing in the ears • Nausea, vomiting • Confusion • Numbness, pain and tingling of the fingers, toes, arms and/or legs, loss of balance
Etoposide	• Infection, especially when white blood cell count is low • Bruising, bleeding • Anemia which may cause tiredness, or may require blood transfusions • Nausea, vomiting • Sores in mouth which may cause difficulty swallowing • Hair loss
Temozolomide	• Headache, seizure • Constipation, nausea, vomiting, diarrhea • Trouble with memory • Difficulty sleeping • Muscle weakness, paralysis, difficulty walking • Dizziness • Tiredness • Hair loss
Vinblastine	• High blood pressure which may cause headaches, dizziness, blurred vision • Infection, especially when white blood cell count is low • Anemia which may cause tiredness, or may require transfusion • Bruising, bleeding • Pain in the bones, jaw, and at the tumor • Constipation • Tiredness • Hair loss
Vincristine	• Headache, jaw pain and/or bone/muscle pain • Numbness and tingling of fingers or toes • Swelling of lower legs • Muscle weakness and difficulty walking • Constipation, which may be severe, as a result of a bowel blockage • Nausea, vomiting, diarrhea • Pain or redness at the site of injection • Hair loss

Table based on data from National Institute of Health: National Cancer Institute. https://ctep.cancer.gov/protocoldevelopment/sideeffects/drugs.htm (32).

Novel and unique targeted therapies, however, have emerged for the treatment of OPG. These targeted therapies continue to evolve and improve and may change the treatment algorithms for OPG in the near future. Patients who fail, are intolerant of, or non-compliant with maximum medical therapy may be candidates for radiotherapy, however, there are significant potential side effects (e.g., impaired intellectual development and endocrine function) in the pediatric population (33, 34). Patients with obstructive hydrocephalus or exophytic lesions may benefit from surgery but in most cases the morbidity (visual loss) and potential mortality (e.g., hypothalamic involvement) precludes surgical resection of OPG. Thus, the optimal treatment for OPG is determined on a case-by-case basis (13, 29).

## Potential and novel treatments

### Immunotherapy and tumor markers

Immunotherapy is a method of tumor treatment that effectively uses or amplifies a patient’s immune system to directly target cancer cells (35). Immunotherapy is a promising option for cancer treatment because of its high degree of specificity, long-lasting effects, and reduction in toxicity and severe side effects (35, 36). Tumor markers are biological molecules that indicate the presence of cancerous growth and can prove helpful in measuring effectiveness of treatment (37). Although few studies have been performed on the role of immunotherapy for OPGs, other studies can provide insight into potential immunotherapy treatments that could be developed specifically for OPGs. A brief summary of the mode of actions for the immunotherapeutic agents discussed can be found in **Table 2**.

**Table 2:** Immunotherapeutic agents modes of action.

Immunotherapeutic Agent	Mode of Action
MEK Inhibitor	Targets MEK, a downstream protein kinase in the MAPK pathway that is often dysregulated and overactive in cancers. Inhibiting MEK-dependent signaling cascades leads to reduction in tumor growth.
BRAF Inhibitor	Targets BRAF, a protein kinase that is part of the MAPK pathway that is dysregulated and overactive in cancers. BRAF mediates signaling from RAS to MEK in the Ras/Raf/MEK/ERK pathway. Inhibiting BRAF kinase interferes with the MAPK pathway of cancer cells and leads to a reduction in tumor growth.
Checkpoint Inhibitor	Cancer cells often dysregulate the normal functioning of the immune system and produce increased checkpoint proteins which cause inactivation of normal T-Cell inflammatory response. Checkpoint inhibitors block checkpoint proteins from binding, allowing T-cell inflammatory response to remain active and attach cancer cells.

### MEK and BRAF inhibitors

MEK and BRAF inhibitors are promising immunotherapy options for children with OPG and have been approved for use in selected pediatric low-grade gliomas (38–40) with specific tumor markers. The MEK inhibitor, selumetinib, was demonstrated to have a 2-year progression-free survival rate of 69% in one study of pediatric patients with progressive or recurrent low grade gliomas (41). Fangusaro et al., reported that up to 96% of recurrent, refractory, or progressive NF1-associated pediatric low grade glioma patients (WHO grades I and II) experienced progression free-survival (PFS) after 24 months of selumetinib (42). PFS was defined as the time period between initial treatment and disease progression or death, or time between initial treatment and last follow-up for patients without progression (42). Additionally, 13 out of 25 patients in this cohort of low grade gliomas had specifically OPG and 10 of the OPG patients had Snellen visual acuity (VA) comparisons in at least one eye prior to treatments (18 valuable eyes at baseline) (42). Among these 18 valuable eyes, there was improvement in vision in 2 eyes and stability (neither improvement or worsening) in 16 eyes. Furthermore, Goldmann perimetry testing comparisons after one year of treatment revealed that of the 10 patients with visual acuity tested, 9 patients had stable visual fields and 1 patient had improvement (42). Other MEK inhibitors including refametinib, binimetinib, trametinib, and cobimetinib have either been approved or are being further developed.

The biochemical pathway targeted in the MEK inhibitors is of interest because the mitogen-activated protein kinase (MAPK) signaling pathway regulates important cellular activities including cell proliferation and often becomes dysregulated by tumor cells (39, 43). The MAPK pathway consists of Ras/Raf/MEK/ERK, and many cancers have been found to be induced by Ras/Raf dysfunction, including sporadic and NF1-associated OPG (39, 44, 45). Selective MEK 1/2 inhibitors block the MAPK pathway and can therefore regulate the proliferation and progression of cancer cells (39).

Raf proteins are also a component of the MAPK pathway that can become dysregulated. BRAF is a gene that encodes the B-Raf protein, which functions as a point of signal transduction in cellular proliferation (46). OPG patients commonly have a point mutation in their BRAF gene known as BRAFV600E and, less commonly, a mutation known as BRAFV600K (47, 48). KIAA1549:BRAF fusion can also occur (47, 48). These genetic alterations can produce an overactive B-Raf protein that dysregulates normal signaling and causes a high degree of cancer cell proliferation. BRAFV600E mutation has been confirmed to result in more aggressive low grade glioma proliferation and resistance to conventional treatment options (48–50). Lassaletta et al. found that the 5-year progression free survival rate in patients with V600E low grade gliomas were less than 50% and 35% for radiation therapy and chemotherapy respectively (analyzed using Kaplan-Meier method and 95% Confidence Intervals) (49). BRAF inhibitors such as vemurafenib, dabrafenib and encorafenib exhibit high specificity for V600 mutated tissues and are associated with positive outcomes; Nobre et al. observed an 80% objective response in BRAF V600E–mutated low grade glioma patients following treatment with dabrafenib or vemurafenib and 53% of patients experienced a greater than 50% reduction in tumor size (48, 51, 52). MEK inhibitors, such as selumetinib, have also been demonstrated to reduce tumor volume in some patients with BRAF mutations; Banerjee et al. found that of the patients with recurrent low grade gliomas that experienced a greater than 50% reduction in tumor size following selumetinib treatment, 4 had BRAF mutations (41). Moreover, combination therapy of MEK inhibitors and BRAF inhibitors (i.e., dabrafenib) are increasingly being utilized in clinical settings. For instance, BRAF and MEK inhibitors combination therapy has been found to increase the objective response rate in patients with melanoma by 15-20% when compared to monotherapy treatments (53). Several projects comparing combination therapy to either MEK inhibitor or BRAF inhibitor monotherapy are currently being performed for different types of tumors; further information should be available in the near future.

From a clinical perspective, a tumor biopsy may be helpful to determine if the OPG harbors the specific BRAF mutation (54). If a BRAF mutation is found, MEK inhibitors and/or BRAF inhibitors may be useful in treatment of the OPG. With these recent advancements, the use of MEK and BRAF inhibitors in treatment of OPG has increased exponentially in the past two years. Research studies are currently evaluating the effects of both MEK inhibitors and BRAF inhibitors as well as the combination therapy in children with OPG.

However, these agents have side effects and several case reports of reversible outer retinal layer separation, retinopathy and uveitis in pediatric patients following MEK or BRAF inhibitor treatment have been observed (55, 56). Children treated with MEK/BRAF inhibitors should be followed with dilated ophthalmic exams to monitor for macular edema and retina breaks or tears. Although the frequency of dilated eye exams necessary is unclear, dilated eye exams typically will be performed every 3 months after beginning MEK/BRAF inhibitor treatment. Time in between appointments can be lengthened appropriately following several unremarkable monitoring exams.

### Checkpoint inhibitors

Checkpoint inhibitors are a major class of immunotherapy treatments that can be used to treat cancer and are being considered in OPG. Tumor cells progress and grow by circumventing the immune system’s regulatory checkpoints thereby preventing T-cells from identifying and attacking tumor cells (36). Checkpoint inhibitors function by blocking the inhibitory checkpoint receptors that are dysregulated by tumor cells thereby inducing a T-cell mediated anti-tumor response (57). Two major checkpoints have been studied in rodent and human models: cytotoxic T lymphocyte-associated antigen 4 (CTLA-4) and programmed cell death protein 1 (PD-1) (58, 59). Much of the current research on checkpoint inhibitors has not focused specifically on OPG. However, discussion of this novel technology is incredibly important as it may serve as a promising future treatment of OPG.

CTLA-4 is an inhibitory T-cell receptor that preferentially binds ligands B7.1/CD80 and B7.2/CD86 expressed on the surface of altered antigen-presenting cells; as a result, T-cell costimulatory receptor CD28 cannot bind these ligands and T-cell proliferation and the immune response is inhibited (60). Anti-CTLA-4 immunotherapy has demonstrated some positive results; tremelimumab has been successful in several clinical trials as a treatment for multiple types of cancer (61). However, in glioblastoma management, anti-CTLA-4 treatment was shown to be no more effective than standard chemotherapy in reducing associated mortality in several different trials (61–64). Further research is needed to determine if anti-CTLA-4 immunotherapy is more effective than chemotherapy for treatment of OPG.

Additionally, CTLA-4 is an important tumor marker that can provide further insight into tumor severity and progression. In a study performed by Liu et al., increased expression of CTLA-4 in patients with low-grade gliomas appeared to be associated with an increased probability of poorer prognosis (62). CTLA-4 can additionally be used to track treatment progress. CTLA-4 should ideally decrease over time with treatment, but there is considerable difficulty in successful drug delivery across the blood-brain barrier which must be addressed (65). Inefficient delivery across the blood-brain barrier is a key challenge that currently prevents checkpoint inhibitors from being utilized in treatment of many more tumors, such as OPG. Novel solutions to overcome this challenge are actively being researched; Galstyan et al. introduced the possibility of using targeted nanoscale immunoconjugates for effective delivery of anti-CTLA-4 across the blood-brain barrier (66). The evidence base continues to expand on the use of these checkpoint inhibitors for OPG, but evidence is not sufficiently robust to make any specific recommendations for treatment indications at this time.

### Bevacizumab-Based chemotherapy treatments

Bevacizumab (Avastin^®^) is an anti-vascular endothelial growth factor (anti-VEGF) monoclonal antibody which is a treatment used in OPG as well as a number of other ophthalmologic retinovascular diseases (e.g., “wet” age-related macular degeneration and proliferative diabetic retinopathy) (67). Bevacizumab is a humanized monoclonal IgG1 antibody that binds to and inhibits VEGF, thereby decreasing angiogenesis and controlling tumor growth and progression (68). VEGF increases angiogenesis and given that OPGs have increased abnormal expression of VEGF, OPG consequently are highly vascularized tumors (69, 70). Gururangan et al. used bevacizumab and irinotecan combination chemotherapy in OPG and reported stabilization in 80% of recurrent low-grade OPG who did not respond to prior traditional chemotherapy and/or radiation therapy (71). Hwang et al. reported an 86% response rate of OPG to bevacizumab-based combination therapy with improvement in visual and neurologic symptoms (72). Moreover, bevacizumab monotherapy has proven to be just as efficacious in improving visual acuity and other symptoms associated with recurrent optic pathway gliomas, while lacking the toxicity associated with irinotecan in combination therapies (72, 73).

Clinically, bevacizumab-based treatments have become increasingly utilized, especially early in treatment if severe vision loss at diagnosis and if chemotherapy or MEK/BRAF inhibitors are not providing adequate treatment. Furthermore, for some patients, a combination of chemotherapy, MEK/BRAF inhibitors, and bevacizumab is useful in treatment of OPG. However, cessation of bevacizumab-based treatments often results in recurrence or progression. Gorsi et al. reported a 91% progression rate following discontinuation of bevacizumab (74). Hwang et al. reported a similar progression rate of about 93% following cessation of bevacizumab treatments (72).

Common side effects from bevacizumab-based treatments include hypertension, fatigue, joint pain, bleeding events, and proteinuria; however, these side effects were transient and reversible following cessation of therapy (38, 71–73, 75, 76).

### Stereotactic radiation techniques

Conventional radiation therapy can be used to treat recurrent OPGs. Stereotactic radiosurgery (SRS) and fractionated stereotactic radiotherapy (SRT) have also been described in OPG. Stereotactic radiosurgery (SRS) is a sophisticated technique that uses the convergence of high-energy radiation beams from many different angles and planes to focus radiation treatment on a specific target, such as an OPG (77). Each radiation beam is not disruptive to neurological tissue development, but convergence of multiple beams in a highly specific location creates DNA mutations that arrest tumor progression (78). SRS refers to a single high-dose radiation session while SRT refers to two to five fractionated sessions of focused radiation (77, 78). SRS and SRT procedures use Image-Guided Radiation Therapy (IGRT) where three-dimensional imaging technologies (MRI, CT, PET) are used to precisely focus the radiation dose to the tumor (77). Fractionated SRT for OPG patients has demonstrated PFS rates of 92% after 3 years, 72% after 5 years, and a 90% survival rate (with or without progression) after 5 years (79).

The Gamma Knife^®^ system has been reported in the treatment of OPG (80). Gamma Knife SRS involves 201 Cobalt-60 beam sources distributed within a spherical cavity that the patient places his/her head into and can treat tumor lesions from 5-40 millimeters (78). El-Shehaby et al. found that single session SRS using the Gamma Knife system resulted in a 90% control rate and 83% PFS rate for OPG (80).

Proton beam therapy is another SRS technique that can be used in treatment of OPG. Proton beams are used in place of photons (as in Gamma Knife) for extremely precise radiation dosing in management of small and irregularly shaped tumors (81). Proton beam therapy has proven to be effective in treatment of brain metastases (82) and low-grade gliomas (83). Indelicato et al. described 174 pediatric patients with low-grade gliomas that were treated with proton therapy which resulted in an 84% PFS rate and a 92% overall survival rate after 5 years (83).

When compared to conventional radiotherapy, stereotactic radiation techniques appear to be favorable with fewer associated toxicities. However, some mild side effects of stereotactic radiation have been reported including nausea, fatigue, vomiting, and pain (84, 85). More information and testing will be required to ensure the long-term efficacy and safety of stereotactic radiation in treatment of OPG.

## Future directions

There are multiple new treatments emerging for OPG, which is increasing the options for individualized treatment of OPG. Surgery for hydrocephalus or for debulking exophytic components of tumor in OPG can be considered but in most cases surgical resection has unacceptable surgical morbidity and mortality. Chemotherapy remains the mainstay for symptomatic or progressive OPG, however, MEK and BRAF inhibitors, bevacizumab, and other immunotherapeutic approaches such as checkpoint inhibitors are showing promise for OPG.

## Author contributions

Category 1: a. Conception and design: AM, MP, CC, and AL. b. Acquisition of data: AM, MP, CC, and AL. c. Analysis and interpretation of data: AM, MP, CC, and AL. Category 2: a. Drafting the manuscript: AM, MP, CC, SB, and AL. b. Revising it for intellectual content: AM, MP, CC, SB, and AL. Category 3: a. Final approval of the completed manuscript: AM, MP, CC, SB, and AL. All named authors meet the International Committee of Medical Journal Editors (ICMJE) criteria for authorship for this article, take responsibility for the integrity of the work as a whole, and have given their approval for this version to be published.

## Funding

Houston Methodist Hospital Education Cost Center: 74002363

## Conflict of interest

The authors declare that the research was conducted in the absence of any commercial or financial relationships that could be construed as a potential conflict of interest.

The reviewer NA-Z declared a shared affiliation with the author AL to the handling editor at the time of review

## Publisher’s note

All claims expressed in this article are solely those of the authors and do not necessarily represent those of their affiliated organizations, or those of the publisher, the editors and the reviewers. Any product that may be evaluated in this article, or claim that may be made by its manufacturer, is not guaranteed or endorsed by the publisher.
