# Supplementary material for: Novel treatments in optic pathway gliomas
Source: Front Ophthalmol (Lausanne). 2022 Sep 29;2:992673. doi: 10.3389/fopht.2022.992673 (PMC11182137; doi:10.3389/fopht.2022.992673)
Supplement: Supplementary file 1 [file Presentation_1.pdf]

Image 1

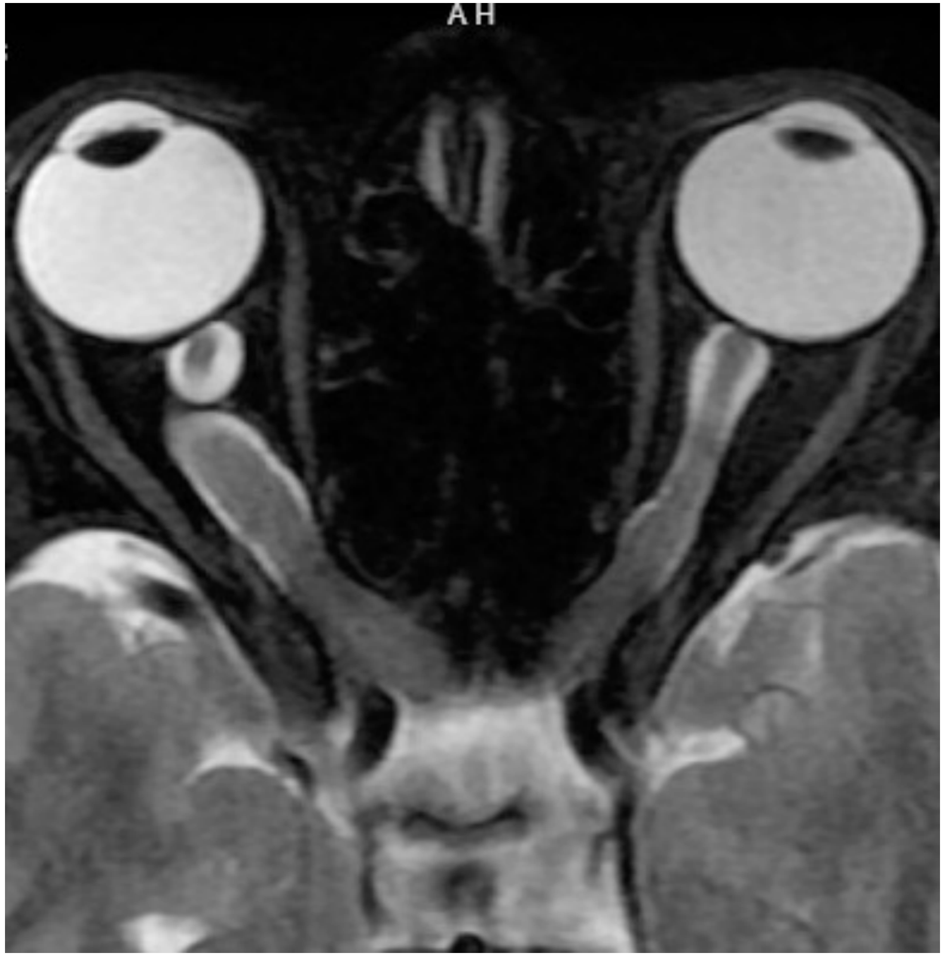

Image 1: Axial T2 with fat saturation: This optic pathway glioma has caused significant enlargement and tortuosity of the right optic nerve with mild enlargement of the left optic nerve.

Image 2 and 3

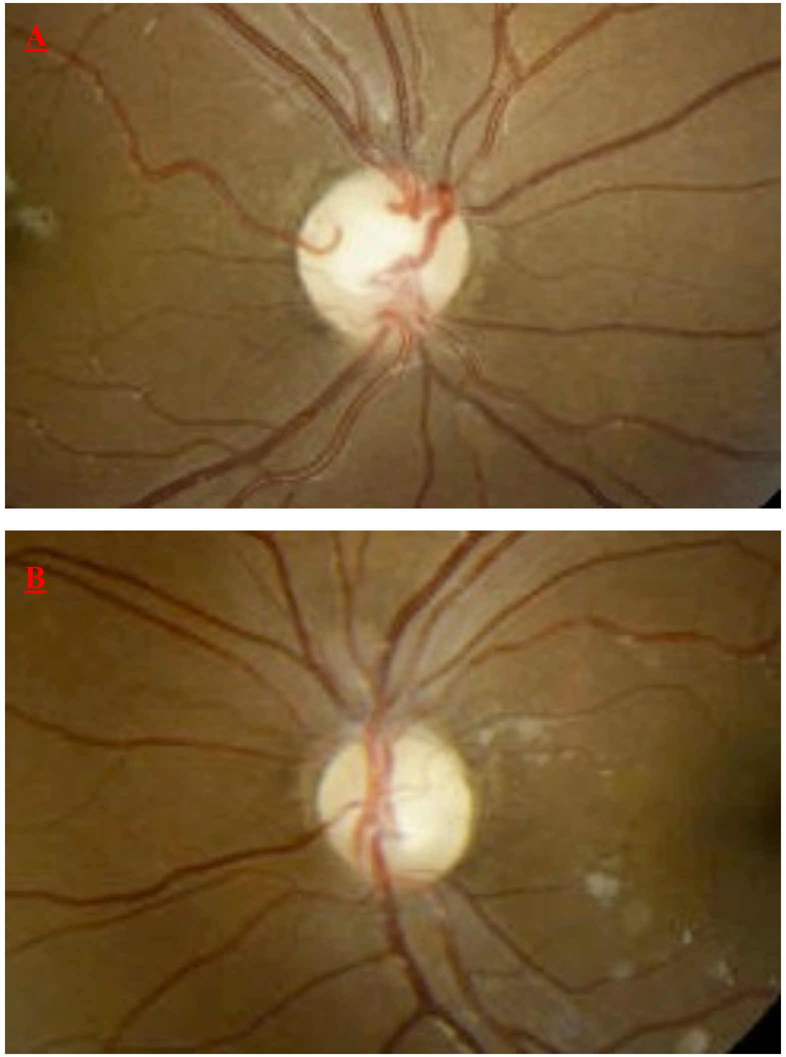

Image 2 & 3: Fundoscopic images demonstrate diffuse pallor of the right optic nerve (A) and left optic nerve (B) with more cupping of the optic nerve suggestive of more long-standing optic atrophy.

Image 4

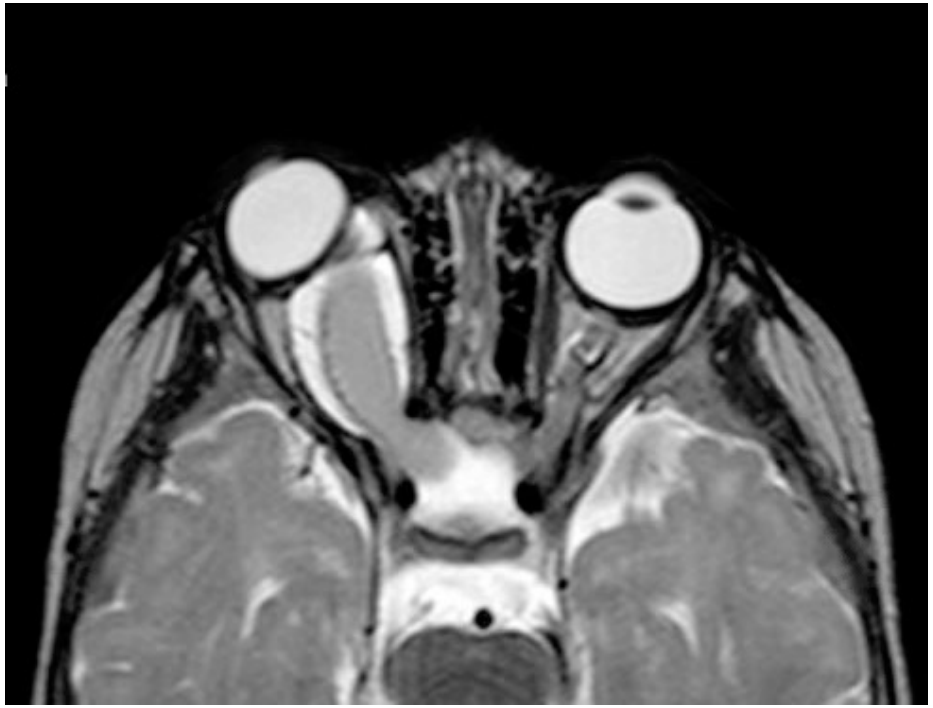

Image 4: Axial T2. An optic pathway glioma in a child without neurofibromatosis type 1 (sporadic case) involving the right optic nerve that is hyperintense compared to the normal left optic nerve. There is enlargement and tortuosity of the optic nerve. Proptosis of the right eye can be seen.

Image 5

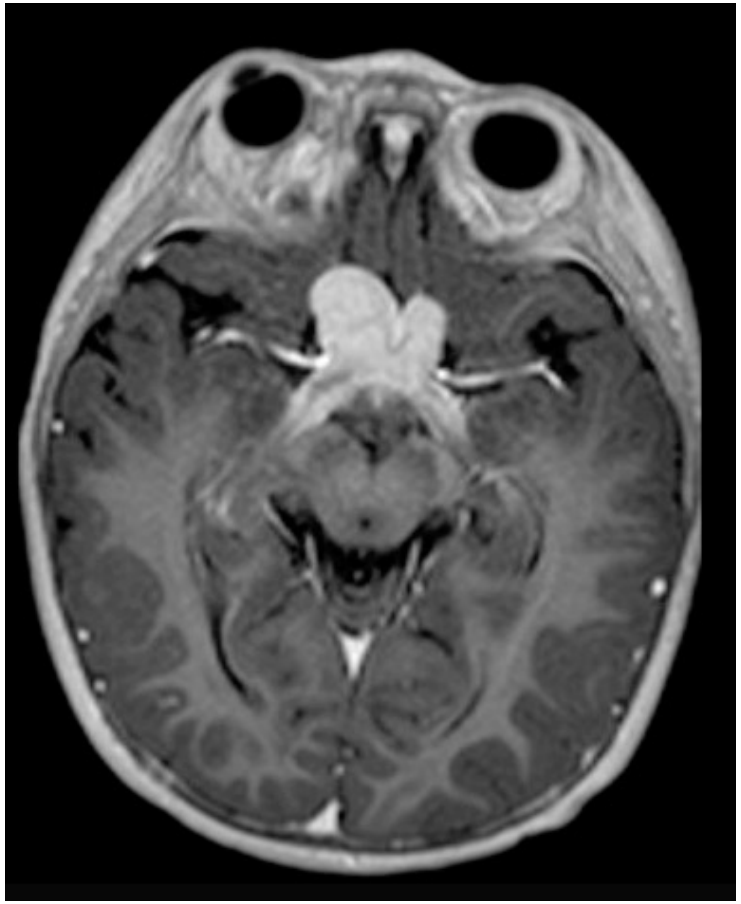

Image 5: Axial T1 with Gadolinium. An optic pathway glioma with expansion and enhancement of the chiasm and bilateral optic tracts in a patient with neurofibromatosis type 1. This patient had right optic nerve glioma (not seen in this axial image) causing proptosis of the right eye.
